# Assessment of Immune and Clinical Response in Patients with Mucosal Leishmaniasis Treated with Pentavalent Antimony and Pentoxifylline

**DOI:** 10.3390/tropicalmed7110383

**Published:** 2022-11-16

**Authors:** Carolina Cincura, Rubia S. Costa, Clara Monica F. De Lima, Jamary Oliveira-Filho, Paulo Novis Rocha, Edgar M. Carvalho, Marcus M. Lessa

**Affiliations:** 1 Serviço de Imunologia, Complexo Hospitalar Universitário Professor Edgard Santos, Universidade Federal da Bahia, Salvador 40110-160, Bahia, Brazil; 2 Serviço de Otorrinolaringologia, Unidade Cérvico-Facial, Complexo Hospitalar Universitário Professor Edgard Santos, Universidade Federal da Bahia, Salvador 40110-160, Bahia, Brazil; 3 Instituto Gonçalo Moniz–IGM–Fiocruz–Bahia, Salvador 40296-710, Bahia, Brazil; 4 Instituto Nacional de Ciência e Tecnologia de Doenças Tropicais–INCT–DT (CNPq/MCT), Salvador 40110-160, Bahia, Brazil; 5 Departamento de Medicina Interna e Apoio Diagnóstico, Faculdade de Medicina da Bahia, Universidade Federal da Bahia, Salvador 40026-010, Bahia, Brazil; 6 Departamento de Cirurgia Experimental e Especialidades Cirúrgicas, Faculdade de Medicina da Bahia, Universidade Federal da Bahia, Salvador 40026-010, Bahia, Brazil

**Keywords:** chemokine CXCL9, chemokine CXCL10, TNF, IFN-γ, disease stage, severity, mucosal leishmaniasis, pentoxifylline, antimony

## Abstract

Mucosal leishmaniasis (ML) is a severe form of tegumentary leishmaniasis associated with a persistent inflammatory response. High levels of TNF, IFN-γ, CXCL9 and CXCL10 are found in ML patients, and the association of pentoxifylline with antimony is more effective in decreasing the healing time in ML patients when compared to antimony alone. The present study aimed to investigate the existence of a correlation between cytokine and chemokine production and ML severity and evaluate the potential value of cytokine and chemokine production as marker of therapeutic response in ML patients. This prospective study included 86 subjects in an area of endemic *Leishmania braziliensis* transmission. Patients diagnosed with ML were classified into clinical stages ranging from I to V according to disease severity. TNF, IFN-γ, CXCL9 and CXCL10 levels were quantified in the supernatant of the mononuclear cell cultures by ELISA before and after treatment with antimony alone or antimony plus pentoxifylline. The median TNF level in the group with mild disease (Stages I–II) was 1064 pg/mL (142–3738 pg/mL), while, in the group with moderate or severe disease (Stages III–V), it was 1941 pg/mL (529–5294 pg/mL) (*p* = 0.008). A direct correlation was observed between ML clinical severity and levels of TNF production (r = 0.44, *p* = 0.007). Patients who were treated with antimony and pentoxifylline healed significantly faster than those treated with antimony alone (52 vs. 77 days, hazard ratio = 0.60; 95% confidence interval = 0.38–0.95, *p* = 0.013). Therapeutic failure was higher in the group that received antimony alone (25% vs. 7%; *p* = 0.041). There was a significant decrease in CXCL9 after therapy of ML in both groups (*p* = 0.013; *p* = 0.043). TNF levels are associated with the severity of mucosal diseases, and pentoxifylline associated with antimony should be the recommended therapy for ML in countries where liposomal amphotericin B is not available.

## 1. Introduction

The leishmaniases are infectious diseases caused by protozoans of the genus Leishmania and are an important public health issue, especially in developing countries [1]. Different clinical forms of American Tegumentary Leishmaniasis (ATL) may arise depending on parasite and host factors [2,3,4]. Nearly 3–5% of patients with cutaneous leishmaniasis (CL) caused by Leishmania braziliensis, the most important causal agent of ATL in Brazil, develop mucosal leishmaniasis (ML) concomitantly with cutaneous lesions, or, in the majority of cases, thereafter [5,6,7]. ML lesions most commonly occur (90%) in the nasal mucosa, generally affecting the cartilaginous nasal septum and anterior portions of the nasal fossa, but may spread to the upper airways and digestive tract (mouth, larynx and pharynx) [8,9]. The nasal mucosal damage ranges from mild nasal nodulations and superficial ulcers to remarkable destruction of the nasal columns. According to its severity, mucosal disease is classified into five stages (I–V) [10]. The standard treatment for ML is pentavalent antimony 20 mg per kg of body weight per day for 30 days) [11]. The association of pentoxifylline, an inhibitor of TNF, plus antimony is more effective than antimony alone, and cures ML patients refractory to antimony [12,13]. However, this documentation has been performed in a limited number of patients. The treatment of ML remains challenging. The number and quality of clinical trials in this severe and much neglected disease is worrisome. 

The Th1 exaggerated immune response seen in CL and ML is characterized by high lymphocyte proliferation response and enhanced production of TNF and IFN-gamma [14,15,16,17]. However, besides contributing to parasite control, the exaggerated productions of theses cytokines are involved in tissue damage and ulcer development [17,18,19,20,21]. For instance, there is a direct correlation between the frequency of CD4 T cells expressing TNF and IFN-γ with the size of the ulcers [18]. Additionally, ML patients produce higher levels of CXCL9 and CXCL10 than CL patients [22]. Both of these chemokines are highly expressed in CL and ML patients’ tissue and participate in the recruitment of T–cells to infected sites [23,24]. There is also evidence in *L*. *braziliensis* infection that CD8 T cells play an important role in the pathogenesis and development of metastatic leishmaniasis lesions and also in the pathogenesis of ML [19,20,25,26]. However, the role of TNF in the severity of ML has not been documented, nor if measurement of TNF or other cytokine levels are markers of response to therapy. The present study aimed to investigate the existence of a correlation between cytokine and chemokine production and ML severity, determine the effect of pentoxifylline on immune response and clinical evolution in ML and evaluate the potential of cytokine and chemokine production as markers of therapeutic response in ML patients. 

## 2. Methods

### 2.1. Subjects

This is an open and prospective study that included 86 patients who were diagnosed with ML, recruited from the Corte de Pedra Health Clinic, an endemic area for *L. braziliensis* located in southeastern Bahia, Brazil, from 2011 to 2015. The exclusion criteria were prior therapy for mucosal disease, diabetes, coinfection with HIV, or chronic kidney, liver or heart disease. Participants were free to participate in the study. All included individuals were volunteers and informed written consent was obtained prior to participation in the study. This study was approved by the Institutional Review Board of the Federal University of Bahia (approval code: 61.2007; approval date: 9 October 2010).

### 2.2. Diagnosis

ML was diagnosed in accordance with the presence of characteristic mucosal lesions in addition to a positive *L braziliensis* PCR [27] parasite isolation or histopathologic findings of ML. 

### 2.3. Clinical Evaluation

A complete otolaryngological examination was performed by an otolaryngology specialist, and each patient’s anterior and posterior nose, as well as the nasopharynx, the oropharynx and larynx was inspected. Before the onset of treatment, patients were classified in stages ranging from I to V according to severity of nasal mucosal damage [10]. Stage I is characterized by nodular lesions without ulcerations, normally along the cartilaginous septum, nasal floor and lateral wall. In Stage II, patients have fine granular lesions, characterized by superficial ulcerations. Stage III is a deep ulceration stage with a more intense tissue granulation and mucosal infiltration. Stage IV is characterized by necrosis of cartilage in the anterior septum, with cartilaginous septum perforation. In stage V, the nasal pyramid is compromised, with alterations of facial features as a consequence of severe tissue destruction. Stages I and II are considered as mild disease and stages III, IV and V as moderate to severe disease.

Individuals were alternated and consecutively allocated in two groups to receive treatment: Group 1 received intravenous antimony (Glucantime^®^, Rhodia Laboratories, Rhone-Poulenc, France) at a dosage of 20 mg per kg of body weight per day for 30 days and Group 2 received antimony at the same dose plus pentoxifylline 400 mg three times a day for 30 days. Monthly clinical evaluations were conducted for at least 6 months after the conclusion of treatment. Cure was defined as complete reepithelization of the mucosal tissue and no evidence of inflammatory activity at day 90 after initiation of therapy. Therapeutic failure was defined as presence of active lesions at day 90.

### 2.4. Cell Culturing and ELISA for Cytokine and Chemokine Analysis

Blood samples used for immunological evaluation were drawn (20 mL) prior to the onset of therapy (Day 0) and between 30 to 40 days after initiation of therapy. Peripheral blood mononuclear cells (PBMC) were isolated from heparin-treated venous blood by Ficoll–Hypaque gradient centrifugation. After washing three times in 0.9% NaCl, cells were re-suspended in RPMI 1640 culture medium (GIBCO BRL, Grand Island, NY, USA) supplemented with 10% human AB serum, 100 IU/mL of penicillin and 100 μg/mL of streptomycin. Cells were plated at 3 × 106 cells/mL in 24-well plates, then stimulated with soluble leishmania antigen (SLA) (5 μg/mL). After incubation for 72 h at 37 °C under 5% CO_2_, cell supernatants were collected and stored at −20 °C. TNF, IFN-γ, CXCL9 and CXCL10 levels were measured by sandwich ELISA (R&D Systems, Minneapolis, MN, USA) and results expressed as pg/mL. The SLA was prepared with an isolate of L. braziliensis as previously described [28]. The ELISA for TNF, IFN, CXCL−9 and CXCL−10 were carried out according to the kit manufacturers’ instructions, with detection sensitivity of 5.5 pg/mL, 5.69 pg/mL, 11.3 pg/mL and 4.46 pg/mL, respectively. All were produced with human recombinant specific for each cytokine, with <0.5% cross–reactivity.

### 2.5. Statistical Analysis

Continuous variables, such as age, were expressed as means ± standard deviation. Cytokine and Chemokine levels were expressed as median and interquartile range. Nonparametric tests were chosen, as the samples did not follow a Gaussian distribution. Variables were compared using Mann-Whitney U test, Fisher exact test, Pearson Chi-Square, Wilcoxon paired test or Spearman correlation as appropriate. A Kaplan-Meier curve, coupled with the log-rank test, was used to evaluate the cure rates. In order to evaluate the effect of clinical stage, baseline cytokine and chemokine levels and the difference between baseline and post-treatment cytokine and chemokine levels on 90-day cure rates, we used Cox regression, adjusted for treatment status. All tests were two-tailed and results were considered significant when *p* < 0.05. Analyses were conducted using Prism (GraphPad Software Inc., San Diego, CA, USA) and SPSS (IBM^®^ SPSS^®^ version 20, Software Inc., Chicago, IL, USA). 

## 3. Results

A total of 86 patients aged 18–70 years with ML were included. Forty four patients were treated with antimony alone (control group), and 42 patients with antimony plus pentoxifylline (pentoxifylline group). All patients exhibited signs of active disease in the nasal mucosa. Patient demographic and clinical features are listed in Table 1. Patients who were treated with antimony or antimony plus pentoxifylline did not differ in any of the baseline characteristics examined. The majority of patients were classified as stage II and III, while fewer patients had mild (I) or the most severe forms of ML (IV–V). Patients who were treated with antimony and pentoxifylline healed significantly faster than those treated with antimony alone (mean ± standard deviation: 52 ± 27 days vs. 77 ± 48; *p* = 0.013). Therapeutic failure, defined as presence of active lesions after 90 days of therapy, was higher in the group that received antimony alone (25% vs. 7%; *p* = 0.041). Therapeutic failure was not associated with severity of disease (*p* > 0.1). Relapses were similar in both groups (14% vs. 12%; *p* = 0.865). 

**Figure 1:**
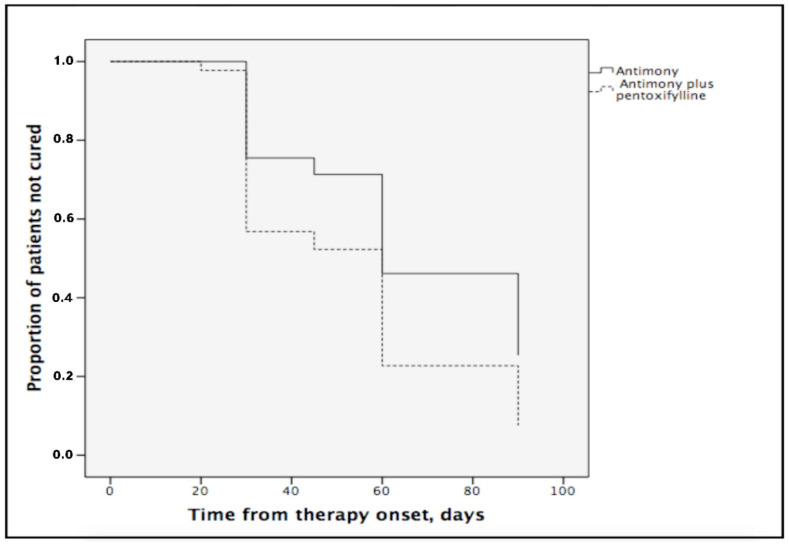
Kaplan-Meier estimates of the proportion of patients not cured with antimony or antimony plus pentoxifylline (Log-rank tzest; *p* = 0.007).

A Kaplan-Meier curve (Figure 1) shows that the 90-day failure rates were higher in the antimony group than in the antimony plus pentoxifylline group (Log-rank test; *p* = 0.007). 

In the Cox regression adjusted for clinical stage, pentoxifylline remained independently associated with lower treatment failure rates (hazard ratio = 0.60; 95% confidence interval = 0.38–0.95, *p* = 0.013). Adverse effects were similar in both groups (five patients in antimony plus pentoxifylline group and three patients in antimony group; *p* = 0.864). The adverse reactions were mild, including nausea, arthralgias, myalgias, abdominal pain and dizziness. No patients in either group discontinued treatment because of these adverse reactions.

**Figure 2:**
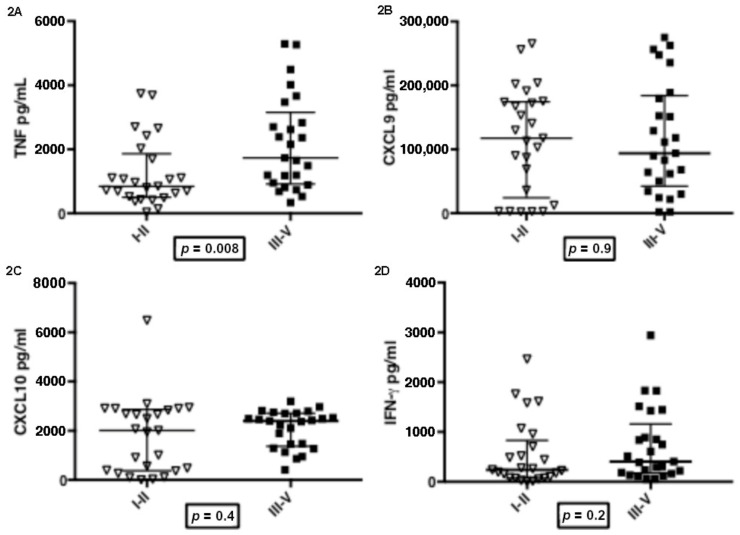
TNF (2A), CXCL9 (2B), CXCL10 (2C), IFN-γ (2D) production and severity of Mucosal Leishmaniasis (stages grouped). I–II: patients with mild disease (*n* = 25); III–V: patients with moderate to severe disease (*n* = 25). Cytokines and Chemokines were measured by ELISA in supernatants of PBMCs stimulated with SLA. Statistics analysis was determined by Mann-Whitney U test.

The immunological evaluations were performed in 50 patients. Before the therapy onset, patients were grouped according to severity of disease: Group 1 (*n* = 25) featured patients classified in stages I and II who presented nodular lesions or superficial ulcerations and Group 2 (*n* = 25) featured patients with more advanced stages (III, IV and V), in which tissue damage was remarkable. The levels of TNF, CXCL9, CXC10 and IFN-γ in both groups are shown in Figure 2. The median TNF level in Group 1 (1064 pg/mL, interquartile range: 142–3738 pg/mL) was significantly higher (*p* = 0.008) than in Group 2 (1941 pg/mL: 529–5294 pg/mL). The median levels of CXCL9, CXCL10 and IFN-γ were not significantly different (*p* > 0.1) between the groups. The median of CXCL9 level in Group 1 was 11,7460 pg/mL (24230–174320 pg/mL), while Group 2 had 93920 pg/mL (42,340–184,210 pg/mL). The median of CXCL10 levels were 2008 pg/mL (376–2866 pg/mL) and 2398 pg/mL (1373–2708 pg/mL), respectively. The median IFN-γ level in Group 1 was 238 pg/mL (76–832 pg/mL), while Group 2 had 402 pg/mL (172–1157 pg/mL). 

**Figure 3:**
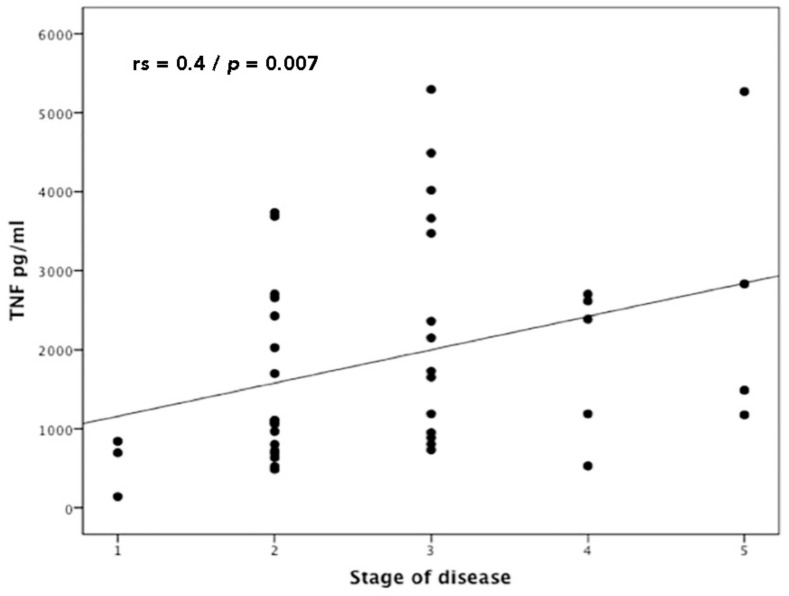
Correlation between TNF production and severity of mucosal leishmaniasis (stage of disease). Statistics analysis was determined by Spearman’s Correlation; ρ {\displaystyle\rho} ρrs = 0.44, *p* = 0.002 (*n* = 50).

A positive correlation was observed between the severity of ML and TNF production (*n* = 50, Spearman’s Correlation; ρ {\displaystyle\rho} ρrs = 0.44, *p* = 0.007) (Figure 3). No significant correlation (*p* > 0.1) was seen between the severity of ML and CXCL9, CXCL10 or IFN-γ production (data not shown). 

**Figure 4:**
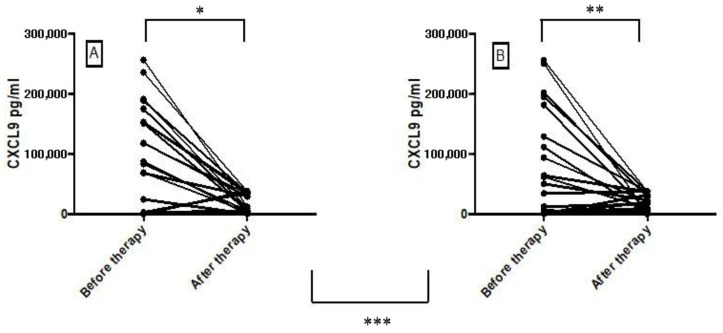
CXCL9 production before (Day 0) and after therapy (Day 30–40) with antimony (**A**) [*n* = 25] and antimony plus pentoxifylline (**B**) [*n* = 25]. The production of CXCL9 was evaluated by ELISA in supernatants of PBMCs stimulated with SLA. Statistical analysis was performed by Wilcoxon matched pairs test (* *p* = 0.013; ** *p* = 0.043) and Mann-Whitney U test (*** *p* = 0.126).

The CXCL9, CXCL10, TNF and IFN-γ levels in the supernatant of PBMCs stimulated with SLA were quantified before (D0) and in D30 of therapy (25 patients treated with antimony alone and 25 patients treated with antimony plus pentoxifylline). There was a significant decrease in CXCL9 after therapy of ML in both groups (Figure 4). There was no significant difference in the levels of TNF, CXCL10 and IFN before therapy and in day 30 after therapy (data not shown). The median level of CXCL9 in the control group was 85,190 pg/mL (25,139–158,650 pg/mL) before therapy and 5539 pg/mL (2427–33,825 pg/mL) after therapy (Wilcoxon matched pairs test; *p* = 0.013). In the pentoxifylline group, the median of CXCL9 was 42,340 pg/mL (3891–14,2300 pg/mL) before therapy and 9360 pg/mL (2742–29,910 pg/mL) after therapy (Wilcoxon matched pairs test; *p* = 0.043). There was no significant difference when compared to the control group and pentoxifylline group (Mann-Whitney U test; *p* = 0.126). No significant increase or reductions (*p* > 0.1) in TNF, CXCL10 or IFN-γ levels were seen after treatment. The changes in cytokines levels after therapy were not associated with therapeutic response or failure. 

## 4. Discussion

ML is a severe clinical form of ATL characterized by mucosal damage that may result in deformities of the face and feeding, breathing and phonation difficulties [8,9,29,30]. Pentavalent antimony is the first-line drug for treatment of ML, but there is a high rate of therapeutic failure [31]. As ML is associated with an exaggerated inflammatory response and TNF plays a key role in the pathology associated with ML, previous studies with small number of patients have shown that pentoxifylline associated with antimony is more effective than antimony alone in the treatment of ML [12]. However, in addition to TNF, others cytokines, such as IFN-γ, CXCL−9 and CXCL−10, are produced in high levels in ML, and the degree of severity of the disease is quite variable, ranging from nodular lesions to septal perforation and destruction of the nasal pyramid. In the present study, we showed that, among the cytokines evaluated, TNF was the only one associated with severity of mucosal disease. We confirmed that pentoxifylline associated with antimony is more effective than antimony alone in the treatment of ML, but the decrease in cytokine production at the end of therapy was not a marker of therapeutic response in ML. 

It is known that antimony therapy is not the best drug for ATL, but, due to the high cost of liposomal amphotericin B, antimony continues to be the first choice drug for treatment of ML in all South and Central American counties [30,31,32]. Antimony therapy associated with pentoxifylline, a TNF inhibitor, has been shown to decrease the healing time in CL and ML patients [12,33], and cure ML patients refractory to antimony therapy [13]. In this prospective study, with a larger number of ML patients when compared to previous studies (86 patients in this study versus 23 patients in pentoxifylline trial for ML) [12], we confirmed that patients who were treated with antimony plus pentoxifylline healed significantly faster than those treated with antimony alone. Moreover, regardless of the severity of the disease, the addition of pentoxifylline remained associated with lower treatment failure rates. Adverse events were mild and similar in both groups without discontinued treatment.

ML predominantly involves the nose. Its severity varies greatly, ranging from the absence of ulcers to the complete destruction of the nasal septum and other nasal structures, which characterizes ML as a heterogeneous disease with five clinical stages [10]. Considering the important role played by a persistent and non–modulated inflammatory response in the development of mucosal disease [2,15], the literature is lacking in studies evaluating the correlation between cytokine and chemokine production and severity of ML. TNF is a cytokine produced mainly by monocytes and macrophages and is produced in high levels in ML [15,16,21]. TNF increases metalloproteinases (MMP-9) production, and high expression of MMP-9 is observed in ML and CL patients [34,35]. There is a direct correlation between the frequency of CD4 T cells expressing TNF and IFN-γ with the size of the ulcers in CL patients [18]. Herein, we demonstrated a clear link between the severity of ML and the increased production of TNF. With respect to chemokines, the present study found high levels of CXCL9 and CXCL10 in ML patients, which is consistent with a few studies that have quantified chemokine levels in ML [22,23], but these chemokines were not found to bear any significant relationship to disease severity. 

A previous study with CL patients showed a decrease in TNF and IFN-γ levels after treatment (day 15 after the onset of therapy), which was more pronounced in the antimony plus pentoxifylline group, and an increase in CXCL9 levels, which was lower in the antimony plus pentoxifylline group [36]. Herein, we found that there was a significant decrease in CXCL9 after therapy of ML (day 30 after the onset of therapy) in both groups. However, there were no significant associations between cytokine and chemokine levels and clinical outcomes. 

We recognize that a limitation of the present study was the small number of cytokines evaluated and the lack of association of severity of ML with other molecules, such as MMP-3, MMP-9 and IL-1β, known to be involved in the pathogenesis of ATL [34,35,37]. Moreover, immunologic evaluations were performed only at two time points on day 0 and day 30. 

The association of pentoxifylline with antimony reduces healing time and therapeutic failure rates, and should be used as the first choice therapy for ML in countries in which liposomal amphotericin B is not regularly used for treatment of ML. We showed that TNF not only participates in the pathogenesis of ML, but is also associated with the severity of mucosal disease. The significant reduction observed in CXCL9 after treatment of the disease and the association of the difference in TNF at the end of treatment with the cure indicate the need for further studies to determine whether the decrease in the production of these cytokines can be considered markers of therapeutic response. in LM.

## Figures and Tables

**Table 1:** Demographic and clinical features of patients with mucosal leishmaniasis.

	Antimony (*n* = 44)	Antimony Plus Pentoxifylline (*n* = 42)	*p*-Value
**Age in years, median (M ± SD)**	36(38 ± 17)	37(42 ± 16)	0.508 ^a^
**Gender: male**	28(64%)	27 (65%)	0.908 ^b^
**LST, median mm (M ± SD)**	17(17 ± 10)	17(16 ± 9)	0.923 ^a^
**Previous or concomitant CL**	33(75%)	34(82%)	0.252 ^b^
**Stage of mucosal disease**			0.231 ^c^
I II III IV V	4(9%) 16(36%) 13(30%) 8(18%) 3(7%)	1(3%) 14(33%) 15(35%) 7(17%) 5(12%)	
**Another location of ML** Oral cavity Pharynx Larynx	4(9%) 8(18%) 1(2%)	3(7%) 5(12%) 1(2%)	0.954 ^b^ 0.558 ^b^ 1.000 ^b^
**Time until Healing, median days (M ± SD)**	60 (77 ± 48)	50 (52 ± 27)	0.013 ^a^
**Therapeutic Failure**	11(25%)	3(7%)	0.041 ^b^
**Relapse**	6(14%)	5(12%)	0.865 ^b^

M: mean/SD: standard deviation; LST: Leishmania skin test result (Largest induration diameter measurement); CL: Cutaneous Leishmaniasis; ML: mucosal lesion; ^a^ Mann-Whitney U test; ^b^ Fisher’s exact test for categorical variables; ^c^ Pearson chi-square.

## Data Availability

Not applicable.
